# MICAL‐L2 potentiates Cdc42‐dependent EGFR stability and promotes gastric cancer cell migration

**DOI:** 10.1111/jcmm.14353

**Published:** 2019-04-29

**Authors:** Pengxiang Min, Shuo Zhao, Lei Liu, Yujie Zhang, Yadong Ma, Xuyang Zhao, Yueyuan Wang, Yixuan Song, Chenchen Zhu, Haonan Jiang, Luo Gu, Jun Du

**Affiliations:** ^1^ Department of Physiology Nanjing Medical University Nanjing Jiangsu China; ^2^ Jiangsu Key Lab of Cancer Biomarkers, Prevention and Treatment, Collaborative Innovation Center for Cancer Personalized Medicine Nanjing Medical University Nanjing Jiangsu China; ^3^ Department of Biochemistry and Molecular Biology Nanjing Medical University Nanjing Jiangsu China; ^4^ School of Basic Medical Sciences Nanjing Medical University Nanjing Jiangsu China

**Keywords:** Cdc42, cell migration, EGFR, gastric cancer, MICAL‐L2

## Abstract

Enhanced migration potential is a common characteristic of cancer cells induced by mechanisms that are incompletely defined. The present study was designed to investigate relationship of a new discovered cytoskeleton regulator MICAL‐L2 and the endogenous epidermal growth factor receptor (EGFR) signalling pathways in gastric cancer cell migration. Increased expression of MICAL‐L2 in gastric cancer cells up‐regulated EGFR protein level, accompanied by the increase of cell migration, whereas silencing MICAL‐L2 down‐regulated EGFR and inhibited cell migration. Expression of MICAL‐L2 was also shown positively correlated with the activation of HSP27/cytoskeleton and HSP27/β‐catenin signalling pathways that provide key mechanisms controlling cell migration. The up‐regulating effect of MICAL‐L2 on EGFR is mediated through a transcription‐independent mechanism that involves inhibiting EGFR protein degradation in lysosome. Further analysis indicated that Cdc42 activation contributed in maintaining the effect of MICAL‐L2 on EGFR stability. Furthermore analysis of clinic specimens revealed increased expression of MICAL‐L2 in carcinoma tissues and a positive correlation between MICAL‐L2 and EGFR expression levels. The above results indicate that MICAL‐L2 potentiates gastric cell migration via inhibiting EGFR degradation in lysosome via a Cdc42‐dependent manner that leads to the activation of EGFR/HSP27 signalling pathways.

## 1 INTRODUCTION

Gastric cancer is the fifth most common cancer worldwide with 952 000 cases diagnosed in 2012.^1^ One of the major reasons for its relatively poor prognosis is that cancer cells spread to other parts of the body without showing any symptoms. Although traditional therapies such as chemotherapy and radiotherapy are being used in clinical practice since a long time. The therapeutic strategies of cancer cell metastasis remain a challenge for the physicians, partly because of our poor understanding of the molecular mechanisms underlying gastric cancer cell migration.

Recent studies have suggested the involvement of some cytoskeleton‐regulating proteins in cell migration. It is already known that the movement of cells is driven by the co‐ordinated rearrangement of actin cytoskeleton. Key regulatory proteins of the actin cytoskeleton such as WASP family proteins, Arp2/3 complex, LIM‐kinase, cofilin linked the formation of invasive protrusions in cancer cells.^2^ MIIP is also thought to contribute in the decreased formation of lamellipodia in endometrial carcinoma cell migration via Rac1.^3^ In recent studies, molecules interacting with CasL (MICALs) were reported to participate in cytoskeleton dynamics.^4^, ^5^, ^6^ The homo sapiens MICAL family consists of three MICAL proteins, (MICAL1‐3) and two MICAL‐L homologues (MICAL‐L1,‐L2). MICAL‐L2, a member of MICAL family, is present abundantly in ovarian cancer tissues.^7^ Silencing of MICAL‐L2 could suppress malignancy of ovarian cancer cells by inhibiting canonical Wnt/β‐catenin signalling and inducing mesenchymal‐epithelial transition.^7^ Furthermore, MICAL‐L2 was also identified preferentially providing ‘law and order' in collective cell migration.^8^ Although the results above suggested that MICAL‐L2 may be involved in cancer cell invasion and metastasis while our knowledge on functions of MICAL‐L2 is limited. Whether and how MICAL‐L2 contributes to gastric cancer cell migration remains largely unknown.

Epidermal growth factor receptor (EGFR), a member of the ErbB family, is considered to be overexpressed in gastric cancer and play role in the development of tumourigenesis.^9^, ^10^ Recent evidence shows that MICAL‐L1 mediates EGFR endocytosis, overexpression of MICAL‐L1 may lead to the accumulation of EGFR in the late endosomal compartment.^11^ MICAL‐L2 has been shown to directly implicate in regulating intracellular transport of multiple cell surface receptors and junctional proteins.^12^, ^13^, ^14^ However, whether MICAL‐L2 regulated EGFR endocytosis and recycling pathway remains unclear. Our data demonstrate that MICAL‐L2 inhibited EGFR degradation in lysosome and promoted stable protein level of EGFR, thus results in maintaining the activation of EGFR pathway and cell migrative potential. Moreover, MICAL‐L2 was shown to maintain the content of EGFR in Cdc42‐dependent manner. Therefore, our findings uncover the contexts in which a recognized cytoskeletal protein MICAL‐L2 functions to keep EGFR content and selective inhibition of MICAL‐L2 may represent a new potential target for gastric cancer metastasis therapy.

## 2 MATERIALS AND METHODS

### 2.1 Ethics statement

All immunohistochemistry assays with human tumour specimens were conducted under the institutional guidelines of Jiangsu Province.

### 2.2 Cell culture

Human gastric cancer cell lines SGC‐7901, BGC‐823 and non‐malignant gastric epithelial cell GES‐1 were bought from the Cell Biology Institute of Chinese Academy of Sciences (Shanghai, China). All cells were maintained in Dulbecco's modified Eagle's medium (DMEM, high glucose) (Hyclone, ThermoScientific, Waltham, MA) supplemented with 10% foetal bovine serum (FBS) (Gibco, Carlsbad, CA), 100 U/mL streptomycin and 100 μg/mL penicillin (Invitrogen, Carlsbad, CA). The cells were incubated at 37°C with 5% CO_2_ in a humidified incubator. Cells were grown on coverslips for immunofluorescence staining and on 6‐well plates (Costar, Corning, NY) for RNA isolation and protein extraction.

### 2.3 Plasmids and siRNAs

The pEGFP‐N1 vectors containing full‐length Cdc42‐Q61L (CA) or Cdc42‐T17N (DN) insert were both saved in this laboratory. Human full‐length MICAL‐L2 cDNA was amplified from pCMV‐SPORT6‐MICAL‐L2 plasmid (YouBio, Hunan, China) using the following primer set, sense: 5′‐CTACCGGACTCAGATCTCGAGCCACCATGGCGGCCATCAGGGC‐3′ and antisense: 5′‐GTACCGTCGACTGCAGAATTCGCTGGGAGGGGCTGCTTTT‐3′. In these primers, XhoI and EcoRI restriction site sequences have been underlined. The PCR products were cloned into the pEGFP‐N1 vector (Clontech, Palo Alto, CA). All constructions were ensured by sequencing. Transfection steps were following the manufacturer's protocols, using Lipofectamine 2000 (Invitrogen, Carlsbad, CA).

The siRNAs were synthesized and purified by China GenePharma Co., and the siRNAs specifically targeting MICAL‐L2 were as follows: #1, 5'‐GGUUCCCACAAAGAGUAUATT‐3′; #2, 5'‐CUCGACGUUUGUGACAACUTT−3′; #3, 5'‐CCAAGUUCCGCUUGUCCAATT‐3′. The transfection of MICAL‐L2 siRNA or control siRNA with Lipofectamine 2000 was performed according to the manufacturer's instruction.

After transfected with plasmid or siRNA for 24 hours, the cells were cultured in starvation medium overnight and then treated with EGF (R&D Systems, Minneapolis, MN), CHX (Sigma, Saint Louis, MO), Erlotinib (APEXBIO, Houston, TX) at the indicated time points.

### 2.4 Cell scratch assays

For scratch assay, a monolayer of cells was cultured in 6‐well plate and then a wound space was made manually with 10 µl pipette tip. After rinsing with PBS, the cell monolayers were treated with indicated stimulator and allowed to migrate for 24 hours. Photographs of wound spaces were taken using microscope (Carl Zeiss Meditec, Jena, Germany).

### 2.5 Transwell assays

Transwell assay was performed with a 24‐well cell culture insert with 8 μm pores. Cells were harvested, washed and suspended in DMEM without FBS and were seeded on the upper chamber with density of 3 × 10^5^/200 μL. Cells were permitted to attach to the membrane for about 30 minutes. The lower chamber was filled with 600 μL DMEM containing 10% FBS. After incubation at 37°C with 5% CO_2_ for 36 hours, the medium in the upper chamber was aspirated out and the cells on the upper side of membrane were removed with a cotton swab. Cells that migrated to the underside of the membrane were stained with 0.1% crystal violet for 5 minutes and visualized and scored under a fluorescence microscope (Carl Zeiss Meditec).

### 2.6 Real‐time quantitative PCR

Total RNA was extracted using Trizol reagent (Invitrogen) and reversely transcribed with HiScript^®^Q RT SuperMix for qPCR (Vazyme, Nanjing, China) according to the protocol. Real‐time PCR analyses were performed with AceQ^®^ qPCR SYBR^® ^Green Master Mix (High ROX Premixed) (Vazyme) on ABI StepOne™ Real‐Time PCR System (Applied Biosystems, Foster City, CA) at the recommended thermal cycling settings: one initial cycle at 95°C for 10 minutes followed by 40 cycles of 15 seconds at 95°C and 60 seconds at 60°C. The gene expression levels were calculated with Rt (2^−ΔΔCT^) values by StepOne Software v2.1 (Applied Biosystems). Primer sequences used in qRT‐PCR were listed: GAPDH: 5′‐CATCAGCAATGCCTCCTGCAC‐3′ (sense) and 5′‐TGAGTCCTTCCACGATACCAAAGTT‐3′ (antisense); MICAL‐L2: 5′‐TGTGGTCCAGAGGAGGAATGA‐3′ (sense) and 5′‐CAGCTCCGGTGGTAAAGCC‐3′ (antisense); EGFR: 5′‐AGGCACGAGTAACAAGCTCAC‐3′ (sense) and 5′‐ATGAGGACATAACCAGCCACC‐3′ (antisense).

### 2.7 Western blotting

Cell lysate was prepared using a total protein extraction buffer (Beyotime, China) and protein concentration was measured using a BCA Protein Assay Kit (Thermo Fisher Scientific, MA) as previously described.^15^ Equal amounts of cellular protein lysates were separated by SDS‐PAGE electrophoresis and transferred to pure nitrocellulose membrane. After blocking with 5% skim milk, the membrane was probed with different specific primary antibodies overnight at 4°C with one of the following primary antibodies. The following antibodies were used: GAPDH (Sigma), MICAL‐L2 (Thermo Fisher Scientific), EGFR (Cell Signaling, Danvers, MA), HSP27 (Cell Signaling), p‐HSP27 (Cell Signaling), Akt (Cell Signaling), p‐Akt (Cell Signaling), GFP (Cell Signaling), Cdc42 (Cell Signaling), Rac1 (Cell Signaling), vimentin (Cell Signaling), E‐cadherin (BD Transduction Laboratories, Franklin Lakes, NJ), N‐cadherin (BD Transduction Laboratories). After incubation with a secondary antibody for 2 hours at room temperature, the bands were visualized with ECL reagent (Millipore, Billerica, MA). Digital images of the positive bands were detected and analysed with Quantity One (Bio‐Rad, Hercules, CA).

### 2.8 Immunofluorescence microscopy

After fixed with 4% paraformaldehyde for 15 minutes, cells grown on glass coverslips were washed with PBS and permeabilized with 0.2% Triton X‐100 for 5 minutes. Then cells were blocked with 1% BSA at room temperature for 1 hour, incubated with primary antibodies against EGFR (Santa Cruz Biotechnology, Santa Cruz, CA) overnight and then with species‐matched secondary antibodies conjugated with Alexa or TRITC‐coupled secondary antibody for 1 hour at room temperature. The nucleus was stained with DAPI (Southern Biotech, Birmingham, AL). The F‐actin was stained with rhodamine‐labelled phalloidin. The immunofluorescence images were acquired with an Olympus BX51microscope (Olympus, Tokyo, Japan) coupled with an Olympus DP70 digital camera.

### 2.9 Pulldown assays

Cells were lysed and Rho GTPase pull‐down assays were performed according to the procedures.^16^, ^17^ Active Cdc42/Rac1 was pulldown by PAK‐CRIB beads. Briefly, protein lysates were centrifuged and supernatant was collected to new tubes containing beads pre‐coupled with PAK‐CRIB and incubated under rotation at 4°C for 30 minutes. Then, the beads were washed and the proteins bound on the beads were separated by SDS‐PAGE. The amounts of active Cdc42 and Rac1 were determined by Western blot analysis with corresponding antibodies.

### 2.10 Cell viability assay

Cell viability was determined by 3‐(4,5‐dimethylthiazol‐2‐yl)‐2,5‐diphenyltetrazolium bromide (MTT) assay as previously described.^18^ In brief, cells at the logarithmic growth phase were collected and seeded in 96‐well tissue culture plates (5×10^3^ cells/well) and transfected with siMICAL‐L2. After culture for 0, 24, 48, 72 hours, 20 μL of MTT (5 mg/mL) was added into each well and the cells were incubated at 37°C in dark for 3 hours. Then, MTT was removed and the dye was solubilized in 150 μL of dimethyl sulphoxide (DMSO). The absorbance at 490 nm was measured using a microplate absorbance reader (Bio‐Tek, Elx800, USA). The percent cell viability was calculated with the following formula: cell viability = (absorbance of the treated well) − (absorbance of the blank well).

### 2.11 Immunohistochemistry

Gastric cancer tissue microarrays were purchased from Outdo biotech (Shanghai, China). Thirty cases of gastric carcinoma samples and their corresponding paracancerous tissue samples were used for immunohistological staining in our study. Briefly, after microwave antigen retrieval, microarray tissues were incubated MICAL‐L2 (Thermo Fisher Scientific), EGFR antibody (Cell Signaling) overnight at 4°C. Following 1 hour incubation with HPR‐conjugated secondary antibody, sections were developed in DAB solution under microscopic observation and counter stained with haematoxylin. Immunohistochemical staining results were taken by using Olympus BX51 microscope. MICAL‐L2 and EGFR immunostaining was scored by assessing the percentage of the number of staining cells and staining intensity, allowing assessment of an immune reactive score (IRS) as described previously.^19^

### 2.12 Statistical analysis

All experiments were repeated at least three times independently. Statistical analysis was performed with the spss statistical software program (Version 19.0; SPSS, Chicago, IL). Data were presented as the means ± SD, the significance of difference in two groups was analysed by Student's *t* test. Values of *P* < 0.05 were considered statistical significance and *P* < 0.01 represents sufficiently statistical significance (two tailed). In immunohistochemistry analysis, Pearson correlation test was used to show the association between MICAL‐L2 and EGFR.

## 3 RESULTS

### 3.1 MICAL‐L2 is up‐regulated and supports EGFR expression in gastric cancer cells

**Figure 1:**
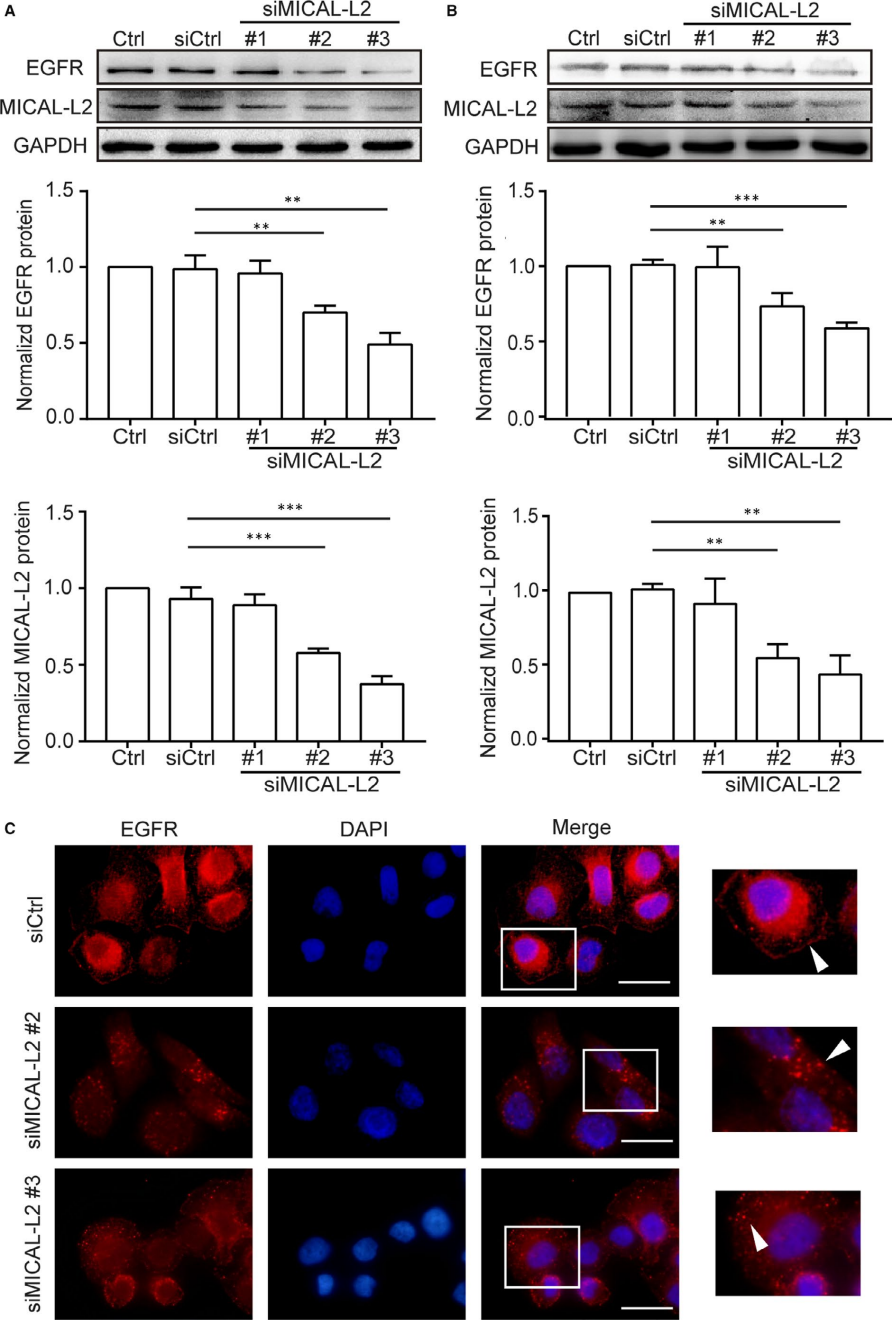
Depletion of MICAL‐L2 inhibits EGFR expression in gastric cancer cells. A, BGC‐823 cells were transfected with control siRNA or siRNA specifically targeting MICAL‐L2 (siMICAL‐L2). Forty‐eight hours later, total protein extracts from cells were analysed for MICAL‐L2 and EGFR expression. Western blot bands corresponding to EGFR and MICAL‐L2 were quantified and normalized against GAPDH. **P < 0.01, ***P < 0.001 in the siMICAL‐L2 cells relative to siRNA control cells. B, SGC‐7901 cells transfected with control siRNA or siMICAL‐L2 were lysed, EGFR and MICAL‐L2 levels were determined by Western blotting assays. **P < 0.01, ***P < 0.001 in the siMICAL‐L2 cells relative to siRNA control cells. C, Representative immunofluorescence images of SGC‐7901 cells transfected with control siRNA or siMICAL‐L2 staining for EGFR. Scale bar, 5 μm

We tested whether MICAL‐L2 plays a role in the EGFR expression in gastric cancer cells and determined the specific mechanisms involved. We first detected the protein levels of MICAL‐L2 in malignant and non‐malignant human gastric epithelial cells by Western blotting. Similar to report by Zhu et al who investigated ovarian cancer cells,^7^ the results showed that MICAL‐L2 as well as EGFR were abundantly expressed in the gastric cancer cell lines SGC‐7901 and BGC‐823 compared to non‐malignant gastric epithelial cell line GES‐1. More MICAL‐L2 and EGFR expressions were found in BGC‐823 cell line that had poorly differentiated feature while less MICAL‐L2 and EGFR were found in moderately differentiated cell line SGC‐7901 (Figure S1). To confirm the role of MICAL‐L2 in regulating EGFR expression, we then silenced MICAL‐L2 expression in BGC‐823 cells with siRNA targeting MICAL‐L2. The cells were lysed and knockdown efficiency was determined by Western blotting. As shown in Figure 1A, siMICAL‐L2 #2 and #3 knocked down MICAL‐L2 expression significantly. siMICAL‐L2 also inhibited the expression of EGFR in those cells. The decrease in the level of EGFR was also observed in MICAL‐L2‐silenced SGC‐7901 cells (Figure 1B). As expected, immunofluorescence staining of EGFR revealed that, in siMICAL‐L2 (#2 and #3)‐transfected SGC‐7901 cells, EGFR mainly distributed in the cytoplasm and its content was decreased when compared with control group (Figure 1C).

### 3.2 MICAL‐L2 positively regulates gastric cancer cell migration

**Figure 2:**
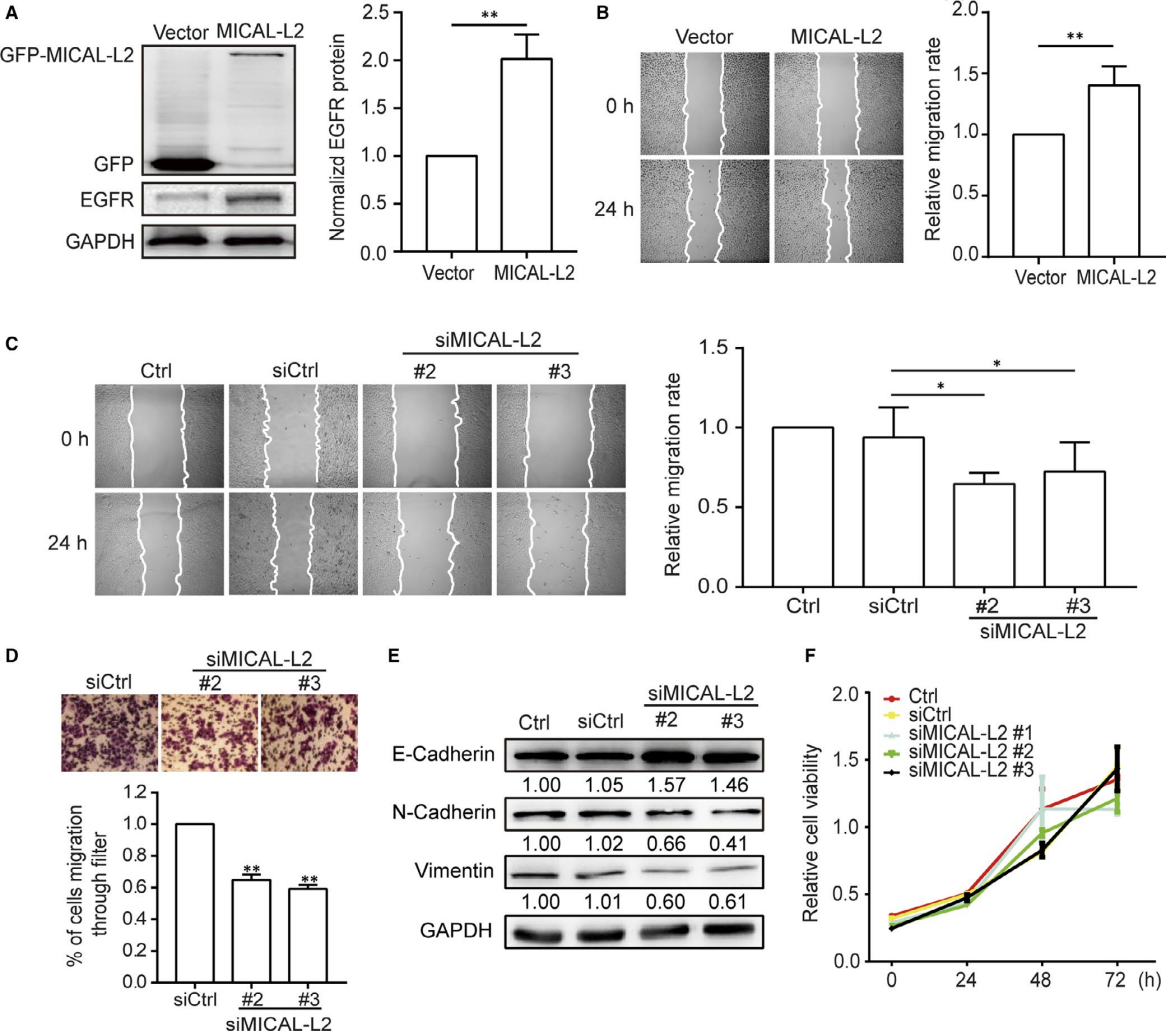
MICAL‐L2 regulates migration of human gastric cancer cells. A, SGC‐7901 cells were transfected with empty vector or MICAL‐L2 plasmids and the total cellular proteins were extracted and analysed for expressions of EGFR by Western blotting assays. Western blot bands corresponding to EGFR was quantified and normalized against GAPDH. **P < 0.01 in the MICAL‐L2 overexpression cells relative to control cells. B, A representative of wound healing assays in SGC‐7901 cells transfected with empty vector or MICAL‐L2 plasmids is presented and the quantification of cell migration rate was performed (n = 8 for each group). **P < 0.01 in the MICAL‐L2 overexpression cells relative to control cells. C, A representative of wound healing assays in BGC‐823 cells transfected with control siRNA or siMICAL‐L2 is presented and the quantification of cell migration rate was performed (n = 8 for each group) *P < 0.05. D, The migration capacity of BGC‐823 cells which transfected with siMICAL‐L2 was also evaluated by transwell assays. **P < 0.01 in the siMICAL‐L2 cells relative to siRNA control cells. E, BGC‐823 cells were transfected with control siRNA or siMICAL‐L2, total protein extracts from cells were analysed by Western blotting and bands corresponding to E‐cadherin, N‐cadherin and vimentin were examined. F, Cell viability of BGC‐823 cells transfected with control siRNA or siMICAL‐L2 was detected by MTT assays

To examine the role of MICAL‐L2 in cell motility regulation, both gain‐ and loss‐of‐function assays were used to alter MICAL‐L2 expression levels in gastric cancer cells. We found that after transfection with MICAL‐L2 plasmids, the EGFR expression (Figure 2A) as well as the migrative potential of SGC‐7901 cells were increased (Figure 2B). By wound healing and transwell assays, we also noticed the migration of the cells transfected with siMICAL‐L2 #2 and #3 were decreased compared with that of control group in BGC‐823 (Figure 2C,D). These results suggested a positive role for MICAL‐L2 in regulating gastric cancer cell migration. We also checked EMT markers expressions, including E‐cadherin, N‐cadherin and vimentin, after depletion of MICAL‐L2 in BGC‐823 cells. The results in Figure 2E showed that silencing of MICAL‐L2 increased E‐cadherin protein level and decreased, N‐cadherin and vimentin levels, implying that MICAL‐L2 might play roles in EMT process of gastric cancer cells. To ensure that the inhibitory effects of MICAL‐L2 were not caused by a proliferation arrest effect, cells were treated with siMICAL‐L2 and their effect on cell viability was determined by MTT assay. The results show that none of the siRNA caused a significant effect on cell viability as compared with control (Figure 2F).

### 3.3 MICAL‐L2 supports EGFR expression by preventing EGFR degradation

**Figure 3:**
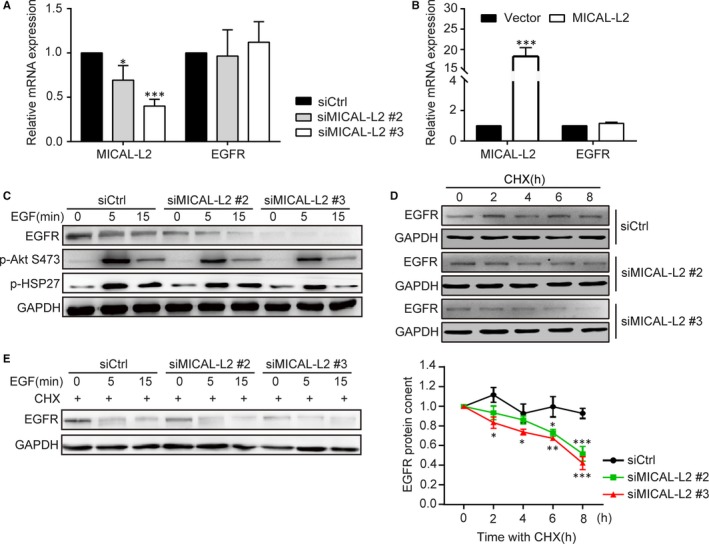
MICAL‐L2 maintains EGFR expression and reduces EGFR degradation. (A, B) The mRNA levels of MICAL‐L2 and EGFR were detected by qPCR in BGC‐823 cells transfected with control siRNA and siMICAL‐L2 (A) and SGC‐7901 cells transfected with empty vectors or MICAL‐L2 plasmids (B). C, BGC‐823 cells transfected with control siRNA or siMICAL‐L2 were in serum‐free media overnight and incubated with EGF (20 ng/mL) for 15 min, protein levels of EGFR, P‐Akt and P‐HSP27 were examined. (D, E) BGC‐823 cells transfected with control siRNA or siMICAL‐L2 were in serum‐free media overnight. After blocking protein synthesis by cycloheximide (CHX, 10 μg/mL), the cells were stimulated with (D) or without (E) EGF (20 ng/mL) for the indicated times. The cells were lysed and EGFR level was determined by Western blotting. GAPDH is used for control. *P < 0.05, **P < 0.01. ***P < 0.001 in the siMICAL‐L2 cells relative to siRNA control cells

To investigate the mechanism of EGFR regulation by MICAL‐L2, cells were treated with siMICAL‐L2 (#2, #3) or MICAL‐L2‐overexpression plasmids, then analysed for *EGFR* mRNA level by qPCR. Whereas SGC‐7901 cells underwent markable reduction or increase in MICAL‐L2, the abundance of *EGFR* mRNA was not altered greatly (Figure 3A,B). Thus, we concluded that instead of transcription‐dependent mechanism, MICAL‐L2 may modulate EGFR expression by suppressing its degradation process. As shown in Figure 3C,D, in BGC‐823 cells, MICAL‐L2 depletion significantly promoted EGFR degradation and EGFR signalling less activation stimulated by EGF when cycloheximide (CHX), a protein synthesis blocker, was existed in media or not. Further, as shown in Figure 3E, after treatment with CHX, silencing of MICAL‐L2 was also shown to accelerate EGFR degradation in BGC‐823 cells without EGF stimulation. Together, these results indicate MICAL‐L2‐mediated EGFR stability was through reducing its degradation process.

### 3.4 MICAL‐L2 prevents lysosome trafficking of EGFR

**Figure 4:**
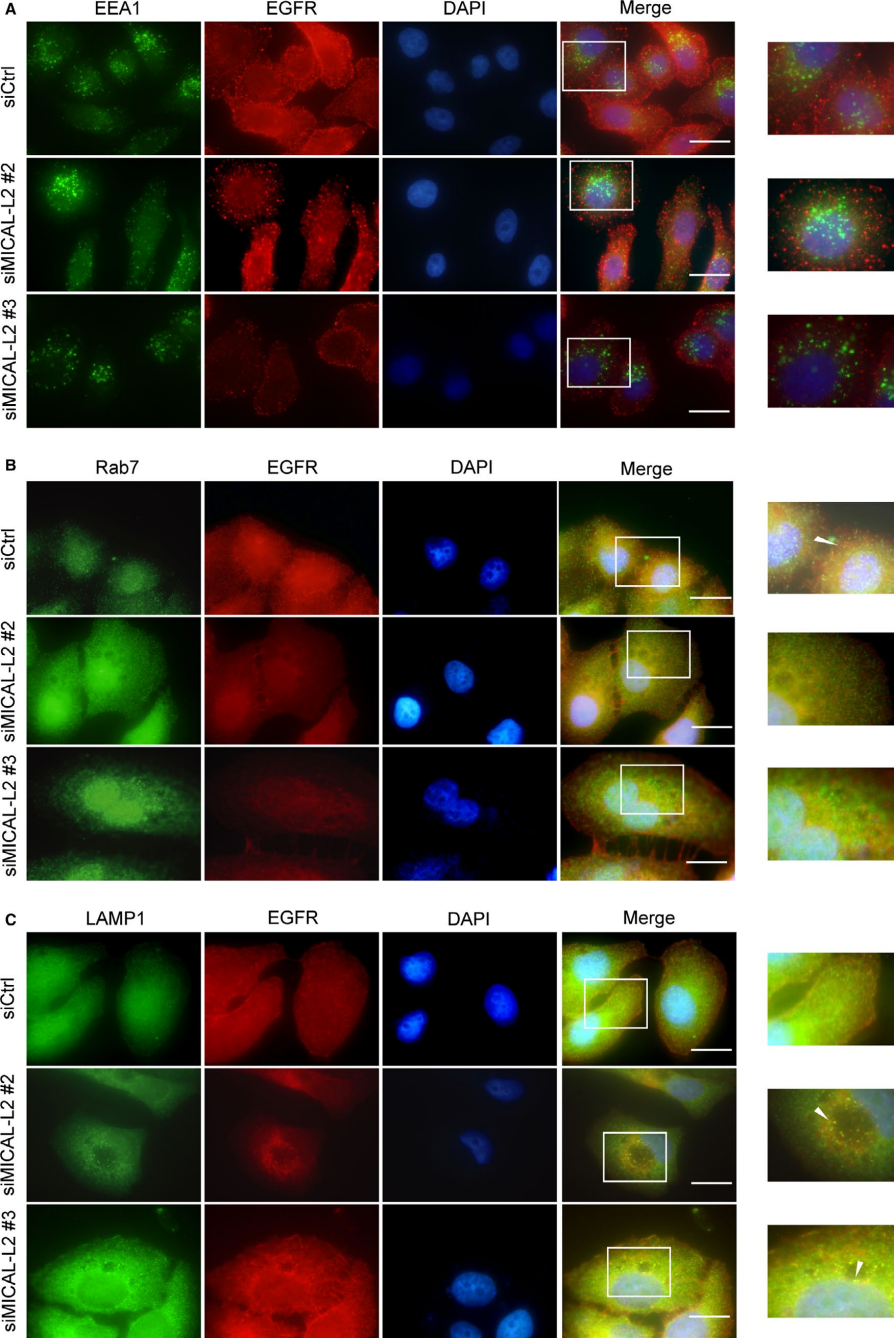
Effect of MICAL‐L2 on EGFR cellular localization. After transfected with control siRNA or siMICAL‐2, SGC‐7901 cells were immunostained by antibodies against EEA1 (A), Rab7 (B) or LAMP1 (C). All endocytic markers are shown in green. EGFR is shown in red. Nuclei (blue) were visualized by DAPI. The yellow colour indicated the colocalization. Scale bar, 5 μm

The degradation of EGFR is regulated by multiple factors. After EGFR binds to ligands, it undergoes dimerization and autophosphorylation. Phosphorylated EGFR then ubiquitinated and enters early and late endosomes in cytoplasm subsequently. Finally, it degrades in lysosomes. The results described above prompted us to determine whether MICAL‐L2 is subcellularly localized to cellular organelles. Immunofluorescence assay showed that EGFR was hardly colocalized with early endosome marker (EEA1), partially colocalized with late endosome (Rab7) and lysosome (LAMP1). We then determined whether EGFR subcellular localization was altered by MICAL‐L2 depletion. As shown in immunofluorescent staining in Figure 4A‐C, MICAL‐L2 depletion led to decreased colocalization of MICAL‐L2 and EGFR in late endosome and increased their colocalization in lysosome, suggesting that knocking down of MICAL‐L2 did not affect its entry into the early endosomes, but promoted EGFR translocation from late endosomes into lysosome. These results suggest that MICAL‐L2 prevents EGFR degradation, possibly by keeping it away from lysosome‐mediated degradation.

### 3.5 EGFR mediates MICAL‐L2‐induced cell migration via HSP27 signalling pathways

**Figure 5:**
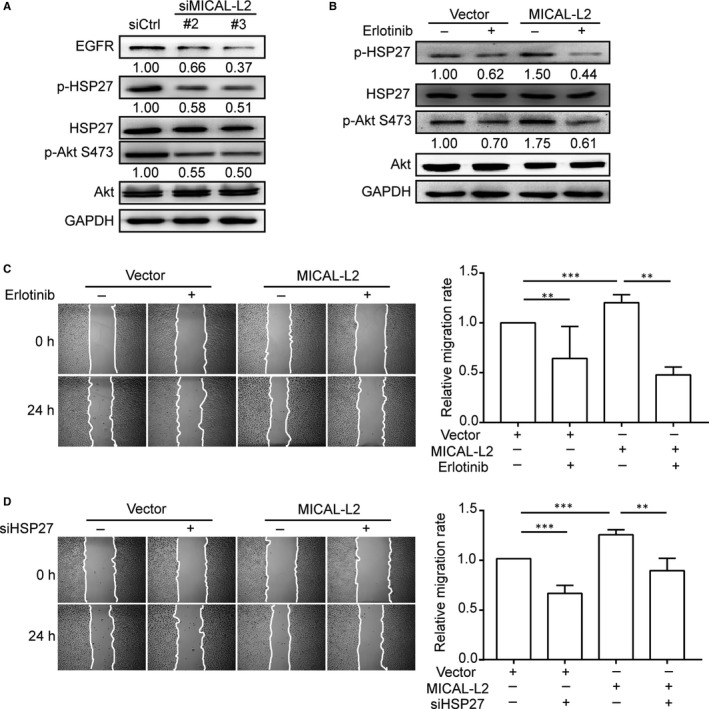
Effect of MICAL‐L2 on EGFR/HSP27 signalling pathways. A, BGC‐823 cells transfected with control siRNA or siMICAL‐L2 were in serum‐free media overnight and protein levels of EGFR, p‐Akt, p‐HSP27 were detected by Western blotting. B, Cells transfected with empty vectors or MICAL‐L2 plasmids were incubated with 1 μmol/L Erlotinib for 24 h. Then proteins extracted from the lysates were subjected to Western blotting to detect the expression of p‐Akt, p‐HSP27. C, SGC‐7901 cells overexpressing MICAL‐L2 were pre‐treated with 1 μmol/L Erlotinib for 24 h, then migration activity of the cells was analysed. D, SGC‐7901 cells overexpressing MICAL‐L2 were pre‐treated with siHSP27, migration activity was analysed. **P < 0.01, ***P < 0.001

We further explored the signalling pathways by which MICAL‐L2 affects gastric cancer cell migration via EGFR activation. Stimulation of EGF leads to HSP27 phosphorylation, then the phosphorylated HSP27 is released from the plus end of the actin filament, these processes are important for actin reorganization and cell motility.^20^, ^21^, ^22^ HSP27 is also required for EGF‐induced Akt phosphorylation and β‐catenin nuclear translocation.^23^ As shown in Figure 5A, silencing of MICAL‐L2 decreased HSP27 and Akt phosphorylation levels. We identified that Akt was a downstream effector of HSP27 in gastric cancer cells (Figure S2A). As shown in Figure S2B, β‐catenin nuclear translocation was also prevented by depletion of MICAL‐L2. The web‐like structure of actin cytoskeleton in cytoplasm was greatly disrupted in MICAL‐L2‐depletion cells (Figure S2C). In contrast, cells transfected MICAL‐L2‐over expression plasmids exhibited corresponding increase in p‐HSP27, p‐Akt levels (Figure 5B). Pre‐treatment with EGFR inhibitor Erlotinib decreased the levels of p‐HSP27 and p‐Akt as well as inhibited the increased migratory activity induced by MICAL‐L2‐overexpression in SGC‐7901 cells (Figure 5B,C). As shown in Figure 5D, the migration rate of SGC‐7901 cells was increased when the cells were transfected with MICAL‐L2 plasmids, which was reduced by siHSP27 pre‐treatment. These results suggest that MICAL‐L2 regulates gastric cancer cell migration by potentiating EGFR‐mediated HSP27 signalling pathways.

### 3.6 MICAL‐L2 regulates EGFR stability via Cdc42

**Figure 6:**
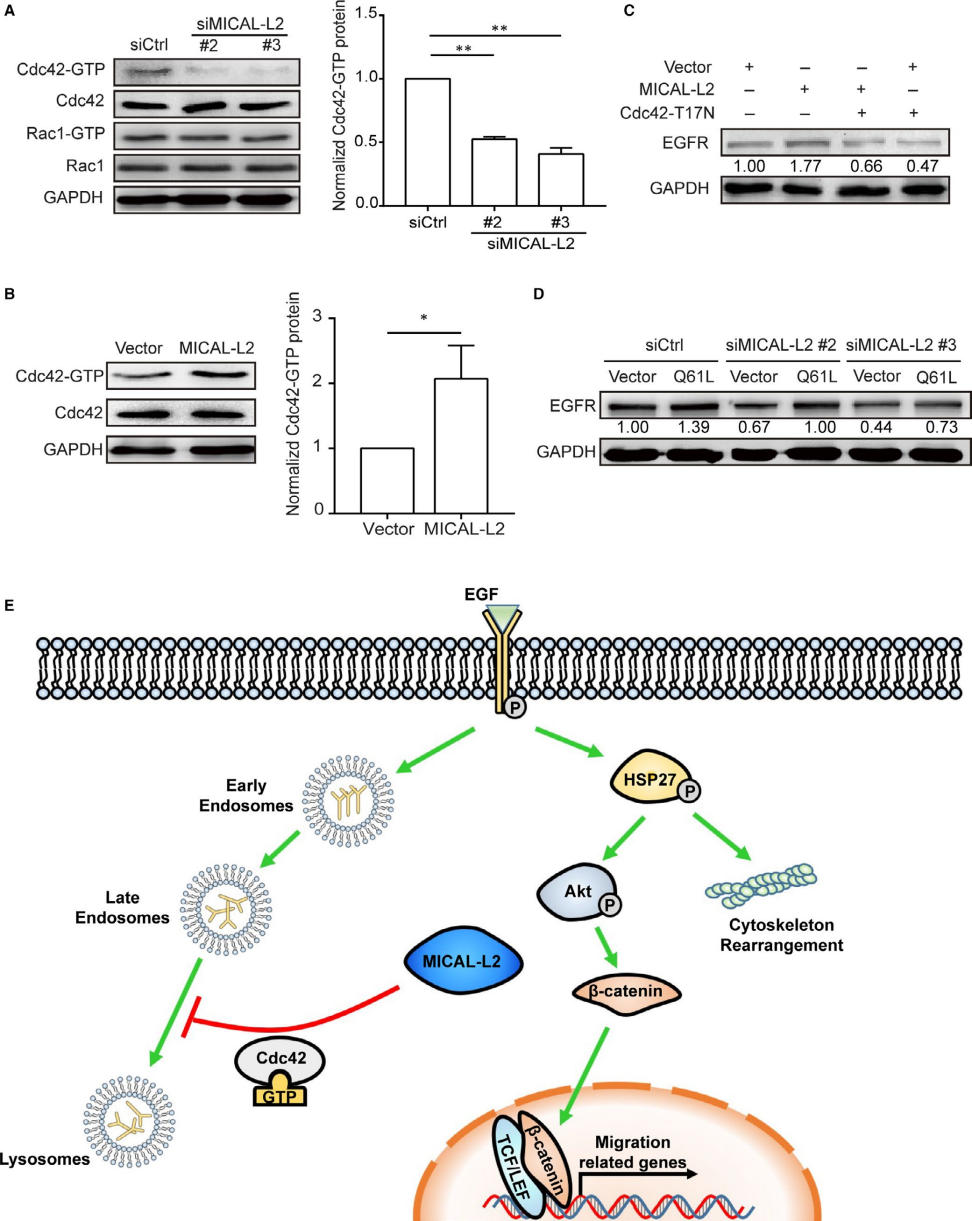
MICAL‐L2 maintains EGFR content by Cdc42 in gastric cancer cells. A, BGC‐823 cells were transfected with siMICAL‐L2 and the activity of Cdc42 and Rac1 was measured by pulldown assays. **P < 0.01. B, SGC‐7901 cells were transfected with MICAL‐L2 plasmids and the activity of Cdc42 was measured by pulldown assays. *P < 0.05. C, MICAL‐L2‐overexpressed SGC‐7901 cells were transfected with Cdc42‐T17N (DN) plasmids and the total cellular proteins were extracted and analysed for EGFR level by Western blotting assays. D, Cells depleting MICAL‐L2 were transfected with Cdc42‐Q61L (CA) and the total cellular proteins were extracted and analysed for EGFR. E, A diagram about the mechanism. MICAL‐L2 potentiates gastric cancer cell migration via supporting EGFR stability and leading to the activation of EGFR downstream HSP27/Akt and Wnt/β‐catenin signalling pathways. MICAL‐L2 maintains EGFR stability, at least in part, through preventing EGFR degradation in lysosome by a Cdc42‐dependent manner

It was reported that Cdc42 plays an important role in the process of internalization and degradation of receptors. To uncover the potential mechanism of EGFR degradation by silencing of MICAL‐L2, we examined Cdc42 activity by pulldown assays in BGC‐823 cells transfected with siMICAL‐L2 (#2, #3) or SGC‐7901 cells transfected with MICAL‐L2 plasmids. As shown in Figure 6A, Cdc42 activity was significantly reduced by MICAL‐L2 knockdown and increased by MICAL‐L2 overexpression (Figure 6B). Cdc42‐T17N (inactive mutant) transfection reversed MICAL‐L2 overexpression‐induced EGFR protein up‐regulation (Figure 6C). Furthermore, we found that EGFR level was elevated when Cdc42‐Q61L (active mutant) plasmids were transfected into the MICAL‐L2‐depleted (#2, #3) BGC‐823 cells (Figure 6D). These results demonstrated that MICAL‐L2 promotes gastric cancer cell migration by Cdc42 activity. In summary, we proposed the mechanisms involved in MICAL‐L2‐regulated gastric cancer cell migration (Figure 6E).

### 3.7 MICAL‐L2 was overexpressed in human gastric cancer samples and correlated with EGFR expression

**Figure 7:**
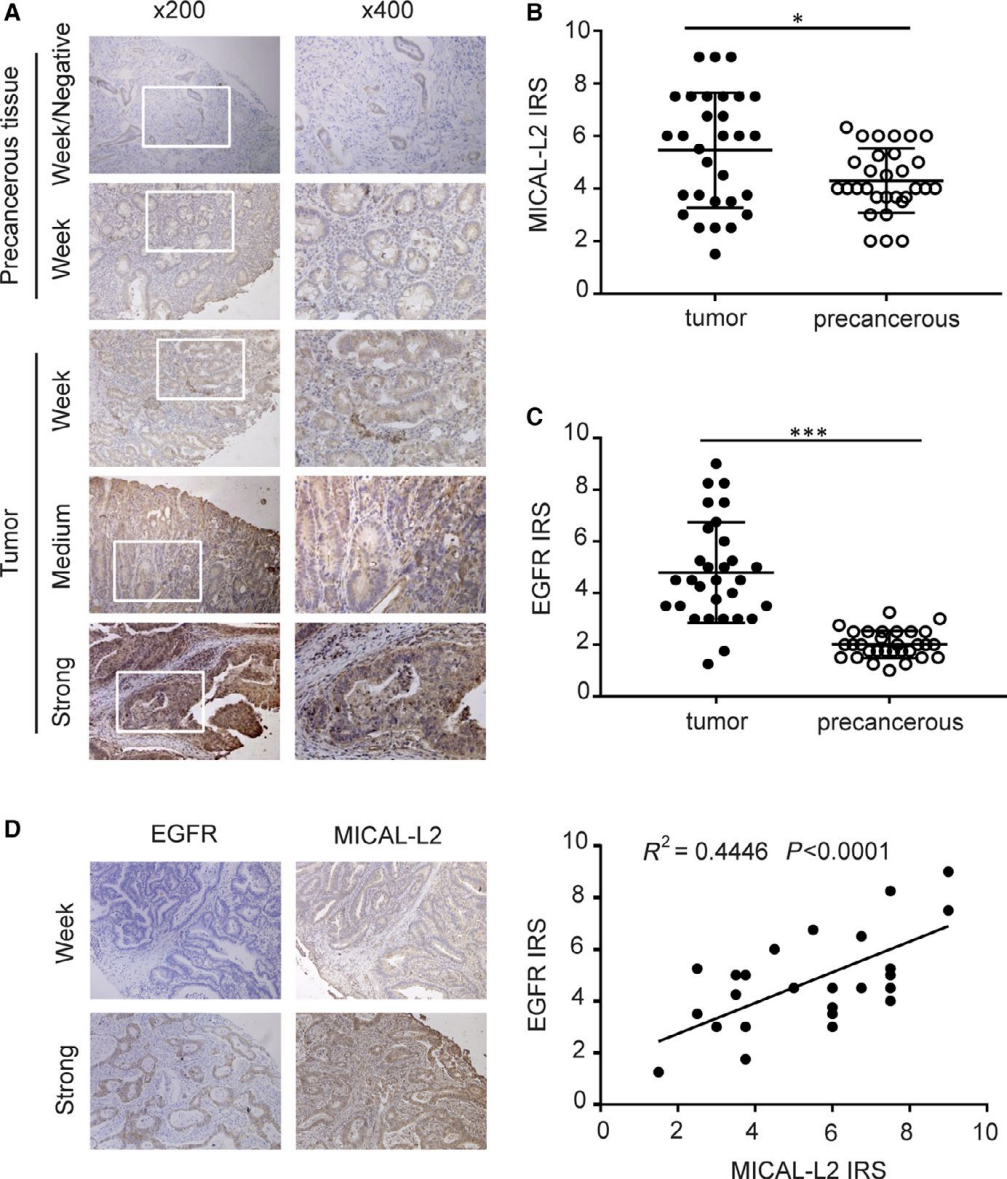
Analysis of MICAL‐L2 and EGFR expressions in gastric cancer tissues. A, Representative images of MICAL‐L2 staining in gastric cancer tissues are shown. The positive staining of MICAL‐L2 is shown in brown colour and the cell nuclei were counterstained with haematoxylin. (B, C) Analysed for MICAL‐L2 and EGFR staining in gastric cancer tissue by IRS scores. D, Using serial sections of the same sample, representative malignant gastric cancer tissue and paracancerous tissue stained for MICAL‐L2 and EGFR are shown. The scatterplot of correlated protein levels between MICAL‐L2 and EGFR in gastric cancer tissue (n = 30) and paracancerous tissue (n = 30). *P < 0.05, ***P < 0.001

In order to explore whether in vitro experimental results were consistent with the pathogenesis of gastric cancer, we examined the expressions of MICAL‐L2 (Figure 7A) and EGFR in gastric cancer tissue and its adjacent tissue by a tissue microarray (30 paired cases). Immunohistochemistry results indicated that both MICAL‐L2 and EGFR were highly expressed in tumour tissues compared with matched paracancerous tissues (Figure 7B,C). Furthermore, MICAL‐L2 expression was correlated with EGFR in these samples (*r* ^2^ = 0.4446, *P* < 0.0001) (Figure 7D). Overall, the clinical data supported our in vitro results revealed a positive link between MICAL‐L2 and EGFR protein expression in gastric cancer.

## 4 DISCUSSION

We demonstrated here that MICAL‐L2 plays an important role in gastric cancer cell migration. Our previous work has reported that MICAL2 is the major regulator of breast cancer cell migration through inhibiting EGFR/P38 signalling activation.^24^ In the current study, we further extended our observation on MICAL‐L2 that lacks flavoprotein monooxygenase (MO) domain and owns C‐terminal domains (CTD) compared with MICAL2.^25^ We showed that gastric cancer cell migrative potential was greatly impaired, which was likely mediated by the knockdown of MICAL‐L2 expression, whereas overexpression of MICAL‐L2 increased cell motility. This result is similar to our previous report that MICAL1, via promoting ROS production by its MO domain, controls breast cancer cell invasive phenotype.^26^ As MICAL‐L2 lacks the MO domain involved in F‐actin oxidation and disassembly, the mechanisms underlying the potentiation of gastric cancer cell migration might be different with other MICAL proteins.

In the present study, we identified a novel link between MICAL‐L2 and EGFR, providing a basis for further exploring the role of EGF/EGFR signalling in MICAL‐L2‐facilitated cancer cell migration. Emerging evidence showed that EGFR amplification has been found in a number of cancers and the constant activation of which may produce uncontrolled cell division.^27^, ^28^, ^29^, ^30^ HSP27 is a notable molecule in EGF/EGFR signalling. We have previously reported the role of p‐HSP27 in MICAL2‐mediated gastric cancer cell migration.^24^ HSP27 is required for EGF‐induced Akt phosphorylation in prostate cancer. Meanwhile, silencing HSP27 decreased EGF‐dependent phosphorylation of β‐catenin on Tyr142, Tyr654 and its nuclear translocation.^23^ Stimulation of EGF also led to HSP27 phosphorylation, then the p‐HSP27 was released from the plus end of the actin filament. These processes are important for actin reorganization and cell motility.^20^, ^21^, ^22^ In this study, we noticed that MICAL‐L2 overexpression‐induced activation of HSP27/cytoskeleton and HSP27/β‐catenin signalling and increased cell migration rate, which were significantly attenuated by the EGFR inhibitor Erlotinib. Whether other EGFR downstream effectors contribute to gastric cell migration requires further exploration. Our results indicate that, at least in part, MICAL‐L2 promotes gastric cancer cell migration via EGFR‐dependent activation of HSP27/cytoskeleton and HSP27/β‐catenin signalling.

Internalization of EGFR is thought to initiate the termination of the signalling from activated EGFR. The fate of internalized EGFR has important consequences for biological cell outputs. The recycling pathway of EGFR favours cell proliferation, whereas the degradative pathway to lysosomes correlates with cellular homoeostasis.^31^, ^32^ Unliganded EGFR can also be internalized but at much slower rate than liganded receptor.^33^We have previously reported that mucin‐like membrane glycoprotein CD24 reduces EGFR internalization and degradation.^34^ In this study, our results first clearly indicated that MICAL‐L2 existence would give support to the stability of EGFR content in gastric cancer cells that play consistent role with the role of two other members of the MICAL family, MICAL3 and MICAL‐L1, in membrane trafficking during cytokinesis.^35^, ^36^ The most important finding here is that MICAL2 identified to be a crucial regulator that links endocytic recycling of EGFR in gastric cancer cells. MICAL‐L2 maintains EGFR level through delaying its degradation, but not by inhibiting its production. To our interest, although silencing MICAL‐L1 was observed contributes to the distribution of internalized EGFR in vesicles spreading throughout the cytoplasm,^11^ our results indicated that silencing MICAL‐L2 is crucial for the accumulation of EGFR in the lysosome compartment. The detailed mechanisms for the difference between the two MICALs are unknown and need further exploration.

Endocytosis of EGFR leads to the deformation of plasma membrane in clathrin‐dependent and ‐independent manner.^37^, ^38^ Local actin polymerization plays an important role in the formation of endocytic carriers.^39^ Rho family is the central link between endocytosis and cytoskeleton. Main members of Rho family include Rac1, RhoA and Cdc42. As we are already familiar that Cdc42 plays an important role in membrane tubulation, vesicle formation and vesicle motility by interacting with other proteins such as N‐WASP and Toca‐1.^40^ It has been reported to facilitate rapid cell surface turnover at the cell leading edge.^41^ Collaboration between Cdc42 and caveolin‐1 was shown to mediate endocytosis of silica‐coated iron oxide nanoparticles in HeLa cells.^42^ In the current study, we noticed that MICAL‐L2 knockdown dramatically reduced Cdc42 activation, while MICAL‐L2 overexpression increased Cdc42 activation, indicating that Cdc42 activation was more likely the target of MICAL‐L2. When we analysed the role of Cdc42 on EGFR stability in gastric cancer cells, we observed that the silencing of Cdc42 impaired MICAL‐L2‐induced EGFR up‐regulation and active form of Cdc42 significantly rescued the attenuated EGFR expression in MICAL‐L2‐depletion cells. So, it is likely that MICAL‐L2 may be able to delay EGFR degradation in a Cdc42‐dependent manner, thereby prolonging EGFR signalling activation and promoting cell migratory properties. Future studies need to investigate the mechanisms in detail about how MICAL‐L2 precisely regulates Cdc42 activation.

In all, these findings demonstrated to the best of our knowledge, the up‐regulation of MICAL‐L2 in gastric cancer cells is attributed to EGFR stability in a Cdc42‐dependent manner. The enhanced EGFR content contributes to activation of its downstream HSP27 signalling and cell migration. Our work establishes the MICAL‐L2 as a key regulator of cell migration and provide promising clues for the development of new therapeutic strategies for treating metastasis in patients with gastric cancer.

## CONFLICT OF INTEREST

The authors confirm that there are no conflicts of interest.

## AUTHORS' CONTRIBUTIONS

JD, PM designed the study. PM, SZ and LL performed the experiments. PM, YZ, YM, XZ, CZ, HJ performed the statistical analysis. JD, PM, YW, YS drafted the manuscript. JD, LG supervised the experimental work. All authors read and approved the final manuscript.

## DATA AVAILABILITY STATEMENT

The datasets used and/or analysed during the current study are available from the corresponding author on reasonable request.
