# Supplementary figures and images for: MICAL‐L2 potentiates Cdc42‐dependent EGFR stability and promotes gastric cancer cell migration
Source: J Cell Mol Med. 2019 Apr 29;23(6):4475–88. doi: 10.1111/jcmm.14353 (PMC6533512; doi:10.1111/jcmm.14353)

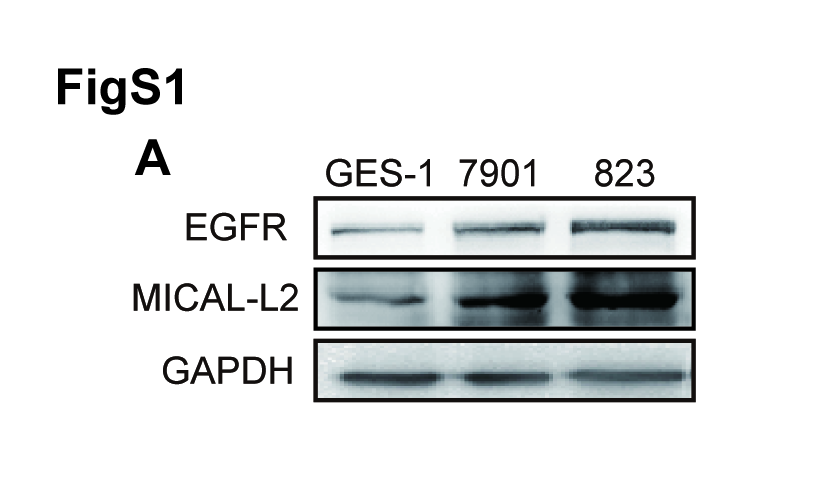

Supplement: Supplementary file 1 [file JCMM-23-4475-s001.tif]

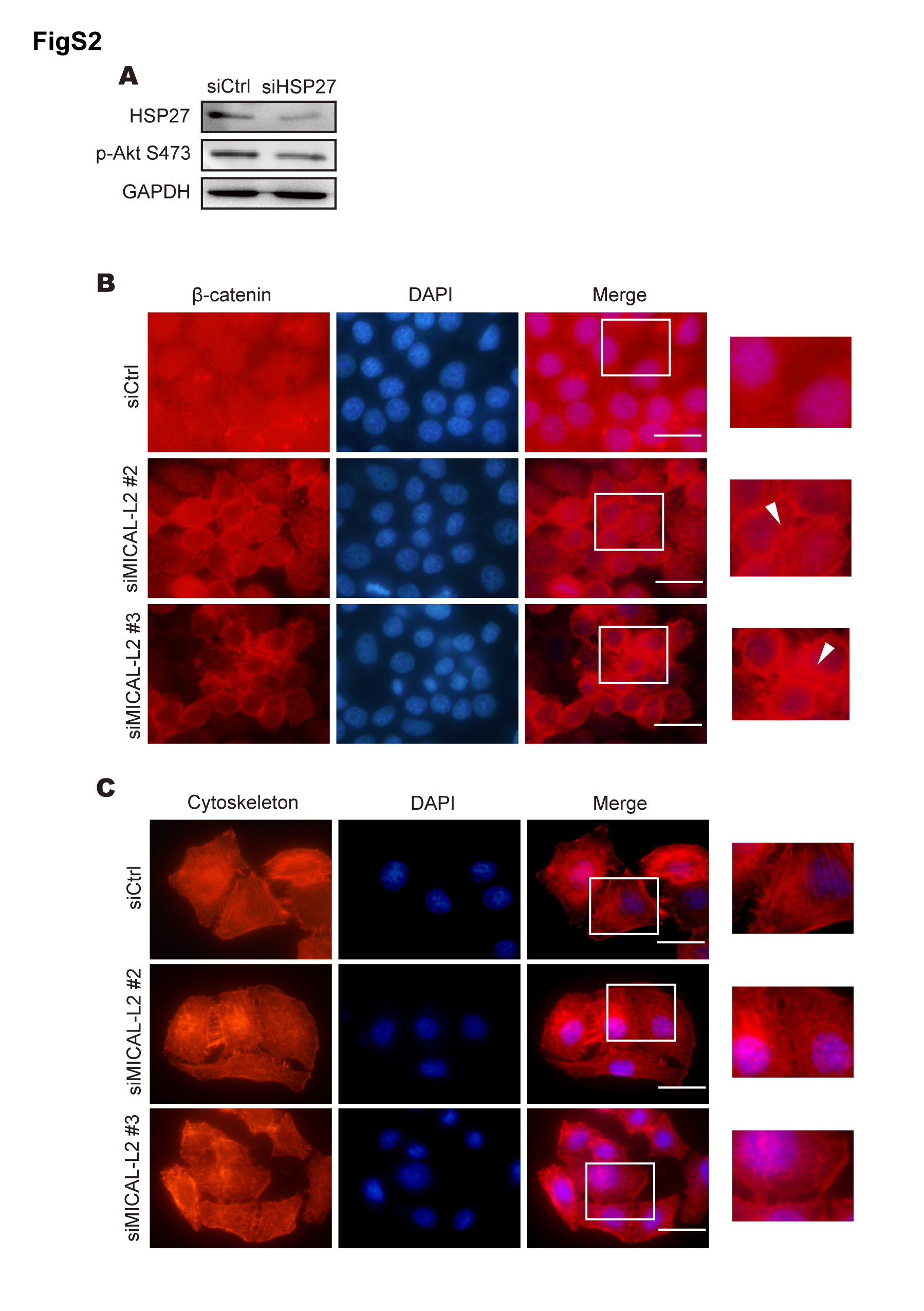

Supplement: Supplementary file 2 [file JCMM-23-4475-s002.tif]
