# Supplementary material for: MICAL‐L2 potentiates Cdc42‐dependent EGFR stability and promotes gastric cancer cell migration
Source: J Cell Mol Med. 2019 Apr 29;23(6):4475–88. doi: 10.1111/jcmm.14353 (PMC6533512; doi:10.1111/jcmm.14353)
Supplement: Supplementary file 3 [file JCMM-23-4475-s003.docx]

**Figure S1. MICAL-L2 and EGFR protein expressions in GES-1, BGC-823 and SGC-7901 cells.**

**Figure S2. Effect of MICAL-L2 depletion on p-HSP27, β-catenin location and cytoskeleton.** (A) BGC-823 cells were transfected with control siRNA or siHSP27. Forty-eight h later, total protein extracts from cells were analyzed for HSP27 and p-Akt level. (B&C) Representative immunofluorescence images of SGC-7901 cells transfected with control siRNA or siMICAL-L2 staining for β-catenin and F-actin. Scale bar, 5μm.
